# Angiographic Aspects of Transarterial Radioembolization: A Comparison of Technical Options to Avoid Extrahepatic Microsphere Depositions

**DOI:** 10.3390/biomedicines12081794

**Published:** 2024-08-07

**Authors:** Peter Etzel, Robert Drescher, Florian Bürckenmeyer, Martin Freesmeyer, Anke Werner

**Affiliations:** 1 Clinic of Nuclear Medicine, Jena University Hospital, Am Klinikum 1, 07747 Jena, Germany; 2 Institute of Diagnostic and Interventional Radiology, Jena University Hospital, Am Klinikum 1, 07747 Jena, Germany

**Keywords:** radioembolization, SIRT, TARE, liver tumor, interventional strategy, extrahepatic microsphere deposition, coiling

## Abstract

The influence of the interventional treatment approach for transarterial radioembolization (TARE) on the incidence of extrahepatic microsphere depositions and to angiographic complications was evaluated. In total, 398 TARE cycles were analyzed. Interventional treatment approaches were classified as single treatment position (TP) with interventional occlusion (IO), multiple TPs without IO, and multiple TPs with IO. Correlations with extrahepatic microsphere depositions, angiographic complications, and periprocedural clinical events were performed. Alternative treatment strategies were evaluated. Applications from multiple TPs could have ensured the safe application of microspheres in 48.2% of cases that were originally performed from a single TP after IO. Extrahepatic microsphere accumulations were detected after 5.2%, 5.3%, and 1.5% of TARE procedures from a single TP without IO, a single TP with IO, and multiple TPs without IO, respectively. Applications from multiple TPs did not increase angiographic complications. During the 30-day follow-up, nausea/vomiting and upper abdominal discomfort were observed more frequently in the group with IO than in the group without IO (7.9%/4.6% and 9.2%/5.9%, respectively). In many TARE procedures, the same target liver can be treated from multiple TPs instead of a single TP, reducing the need for the interventional occlusion of aberrant arteries and potential extrahepatic microsphere depositions.

## 1. Introduction

Malignant changes in the liver can be of primary (hepatocellular carcinoma or cholangiocellular carcinoma) or secondary origin (e.g., metastases of a colorectal carcinoma or breast carcinoma). Often, these diseases present with non-specific symptoms, such as weight loss, inappetence, ascites, right-sided abdominal pain, or liver failure, and are recognized only in advanced stages.

Transarterial radioembolization (TARE) is an established, targeted interventional procedure in the therapeutic algorithm for the treatment of liver tumors. Since the first reports of yttrium-90 application in 1965, TARE has evolved into an increasingly sophisticated angiographic technique [1]. Whereas entire livers were treated in the past, nowadays, a lobar approach is used, or only the tumor-affected liver segments are treated. For this purpose, selective dose planning and reproducible angiographic microcatheter positioning are of great importance. Important challenges include how to proceed with variant arteries and to further avoid extrahepatic microsphere depositions, which can potentially lead to the unfeasibility of TARE or may result in potentially life-threatening complications. Traditionally, aberrant vessels were occluded; however, another treatment strategy has evolved with the application of multiple therapy positions (TPs) distal to an aberrant vessel.

The aims of this study are to evaluate the influence of the complexity of the intervention (i.e., number of catheter positions, vascular occlusions) on the incidence of extrahepatic microsphere depositions and adverse events, to analyze the flexibility of the interventional strategies applied for TARE, and to formulate recommendations for the technical avoidance of extrahepatic microsphere depositions when extrahepatic vessels are identified.

## 2. Materials and Methods

### 2.1. Patient Cohort

This study was approved by our Institutional Review Board, reg. no. 2020-1908. Patient inclusion was consecutive. Over a period of 8 years and 7 months, 270 patients (220 males and 50 females; mean age 65.3 ± 9.7 years) underwent TARE therapy for the treatment of HCC (65.2%), CCC (6.7%), or liver metastases (28.1%). In total, 438 TARE planning procedures (evaluations) and 398 TARE treatments were performed. Overall, 128 patients (32.1%) underwent multiple TARE treatment procedures (range 2–4 procedures).

### 2.2. TARE Procedural Data, Image Analysis and Follow-Up

TARE evaluations to determine activity distribution in and outside the liver and to calculate the lung shunt fraction were performed with ^99m^Tc-labeled HSA B20 microspheres (ROTOP Pharmaka GmbH, Dresden, Germany). TARE treatments were performed with ^90^Y-containing resin microspheres (SirSpheres; Sirtex Medical, Woburn, MA, USA), ^90^Y-containing glass microspheres (TheraSphere; Boston Scientific, Marlborough, MA, USA), or ^166^Ho-containing poly-L-lactic acid (PLLA) microspheres (QuiremSpheres; Terumo, Leuven, Belgium). A database was created containing the radiological and nuclear medicine findings on TARE evaluations and treatments. All TARE-related forms of imaging, including pre-therapeutic CT and/or MRI, TARE planning and treatment digital subtraction angiography (DSA), SPECT/CT, and PET/CT images, were reviewed by a radiologist and a nuclear medicine specialist with more than 6 years experience in TARE therapy.

The hepatic vascular anatomy, according to Michels’ classification, treated liver lobes/segments, microcatheter positions in the liver vasculature for microsphere application (treatment positions, TP), and the type of injected microspheres were noted. The use and target vessel of interventional occlusion (IO) techniques were analyzed and classified as therapeutic or prophylactic IO (tIO or pIO) based on the relation of the TP for microsphere application to the occluded vessel. An IO of a vessel proximal to the aspired TP was considered prophylactic, i.e., only important when a microsphere backflow occurred. An IO of a vessel distal to the aspired TP was considered therapeutic to avoid an otherwise inadvertent microsphere inflow.

Extrahepatic microsphere depositions were localized, and the intensity of extrahepatic enhancement measured on SPECT was compared with tracer intensity in the treated, non-tumor-bearing liver and classified visually as above or below the activity in the non-tumor target liver. Factors limiting the desired application of microspheres and peri-interventional complications occurring during the angiographic procedures and during a 30-day follow-up period were noted and classified according to CTCAE version 5.0 [2].

Interventional strategies and technical aspects were correlated with the incidence of extrahepatic microsphere depositions and adverse events. Microsphere deposition intensity was categorized on SPECT/CT or PET/CT as above or below the activity concentration in the non-tumor target liver. The therapists’ handling of each extrahepatic deposition with the identification of the responsible vessel, change in therapy position, or acceptance in cases of limited manifestations was recorded. Periinterventional adverse events were attributed to the angiography procedure with TARE-specific steps (injection of radioactive substances or embolization effects) or the follow-up period.

### 2.3. TARE Interventional Strategies

By reviewing DSA images, post-procedural SPECT and PET/CT images, and interventional reports, TARE treatments with the simplest interventional strategy were identified: the liver target volume was flooded with microspheres from a single TP, and no therapeutic IO of extrahepatic branches was performed. In the remaining TARE treatments, interventional strategies used to avoid extrahepatic microsphere depositions while still covering the intended liver target volume were identified and classified into the following three categories:

- Single TP with tIO: the application of the microspheres from a single TP after the IO of an extrahepatic branch distal to the TP;
- Multiple TPs without tIO: application of the microspheres from multiple TPs without IO;
- Multiple TPs with tIO: the application of microspheres from multiple TPs after the IO of an extrahepatic branch distal to the TP.

**Figure 1:**
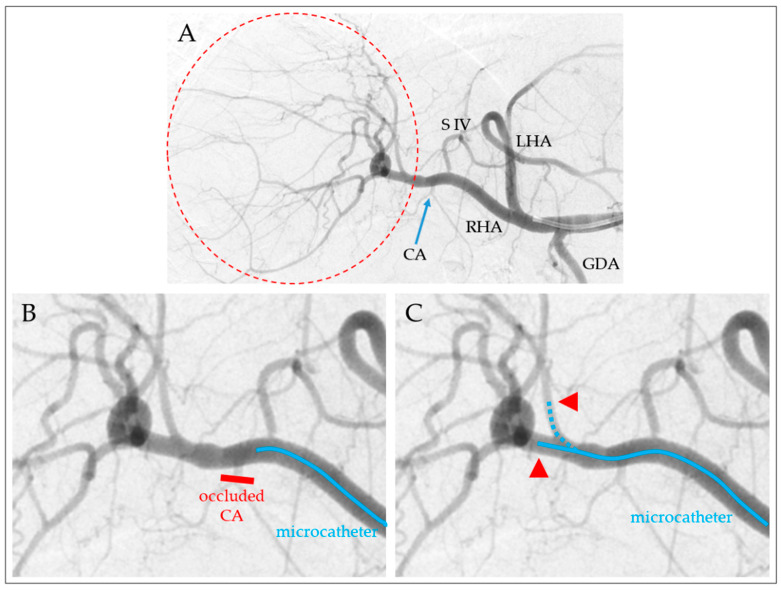
The choice of interventional strategies for the TARE of the right liver lobe (**A**); dashed circle: target area). Michels type I vascular anatomy, with an accessory segment IV branch. For TARE, the cystic artery (CA) can be interventionally occluded, followed by proximal microsphere application (**B**). Alternatively, microsphere application can be performed from two more distal therapeutic positions ((**C**), arrowheads) without CA occlusion. (RHA/LHA right/left hepatic arteries; GDA, gastroduodenal artery).

In the second step, based on the angiographic images, it was assessed if an alternative interventional strategy could have been applied to achieve the same vascular and liver target volume coverage by performing TARE from multiple (up to three) instead of a single TP to reduce the number of tIOs, or to perform more tIOs of the extrahepatic vessels to enable microsphere application from a single instead of multiple TPs (Figure 1).

### 2.4. Statistical Analyses

Statistic data analysis, the graphical presentation of results, and the calculation of evidence were performed using the statistical software R (version 12.08.2021, R Foundation, Vienna, Switzerland). Fisher’s Exact Test was applied to evaluate the differences in the individual study results. A *p* value of <0.05 was considered significant. Categorical characteristics were described using absolute and relative frequencies, and metric characteristics were represented using the mean and standard deviation or the median with minimum and maximum.

## 3. Results

### 3.1. TARE Procedural Data and Periprocedural Adverse Events

Michels type I represented the most common hepatic vascular anatomy (70.7%), followed by types V, III, and VI (11.8%, 5.2%, and 4.4%, respectively) [3].

IO was conducted in 76 TARE treatments (19.1%). The most common intention was to avoid extrahepatic microsphere accumulation in the gallbladder (*n* = 63, 79.7%; Table 1). IOs were performed with coils (86.8%), vascular plugs (9.2%%), and foam (3.9%).

^90^Y-glass, ^90^Y-resin, and ^166^Ho-PLLA microspheres were used in 203 (51.0%), 188 (47.3%), and 7 (1.7%) of TARE treatments, respectively. In 74 TARE treatments, the application was performed from more than one TP, in 73 TARE treatments (18.3%) from two TPs, and in 1 procedure from three TP. Applications from multiple TPs were performed to avoid extrahepatic microsphere accumulation or backflow, and for the treatment of target liver volumes supplied by different arteries (Table 2), the most frequently used TPs were in the right and the left hepatic arteries (41.1% and 37.0%, respectively).

### 3.2. Adverse Events and Technical Limitations

The rate of angiographic adverse events was 2.3% and 1.8% for the TARE evaluation and treatment, respectively (Table 3). The most frequent adverse events encountered were inguinal hematoma, small vascular dissections, and coil displacement. The most frequent technical limitations were the reversible vasospasms of intrahepatic arteries during probing and the unsuccessful tIO of cystic arteries. Therapy discontinuation was not necessary. Small pseudoaneurysms of intrahepatic arteries were detected in two patients; in one patient, this probably resulted from the angiographic procedure of the patient’s second TARE evaluation but did not affect the microsphere application strategy. Angiographic complications were of mild or moderate severity (CTCAE < 3).

The frequency of adverse events and technical limitations other than inguinal hematoma did not differ significantly between interventional strategies (single TP without/with tIO, multiple TPs without/with tIOs at 2.2%, 1.9%, 1.5%, and 0%, respectively). During follow-up, adverse events were noted in 87 patients (21.9%), including, most frequently, abdominal pain (26 patients), nausea/vomiting (21 patients), fatigue (14 patients), fever (11 patients), and tachycardia (10 patients).

The most common postinterventional symptoms in the 30-day follow-up period after TARE treatment included upper abdominal discomfort (6.5%), nausea and vomiting (5.3%), fatigue (3.5%), and fever (2.8%). Follow-up adverse events occurred after 17 of 76 treatments with IO (22.4%) and after 70/322 treatments without IO (21.7%). In these groups, nausea and vomiting occurred after 7.9% and 4.6%, and upper abdominal discomfort occurred after 9.2% and 5.9% of TARE therapies, respectively. In total, 11 patients (2.8%) suffered events with CTCAE grade 3 or higher, including fatigue, hypertension/tachycardia (3 patients), pulmonary edema, esophageal variceal bleeding, esophageal ulceration, cholangitis, pancreatitis, acute renal failure, and hepatorenal syndrome.

### 3.3. Extrahepatic Microspheres Depositions

After TARE therapy, extrahepatic microsphere depositions were detected in 18 patients (4.5%; Table 4). In nine patients, the accumulations were already visible during TARE evaluation. Gallbladder and duodenum accounted for more than 2/3 of the microsphere depositions. Three patients had related clinical symptoms without correlation on follow-up-imaging (accumulation in gall bladder: abdominal pain; accumulation in gastric wall: nausea/vomiting; accumulation in connective tissue and duodenum: pain/nausea/tachycardia). One patient with focal microsphere deposition at the distal esophagus developed ulceration, pain, and dysphagia. The events of esophageal variceal hemorrhage, cholecystitis, and pancreatitis were not associated with extrahepatic microsphere accumulation in the corresponding organs.

### 3.4. Interventional Strategies for TARE Treatment Procedures

In total, 271 TARE treatments (68.1%) were performed with the simplest strategy: from a single TP without the tIO of extrahepatic arteries. In total, 127 TARE treatments (31.9%) were performed from multiple TPs and/or after tIOs were classified as single TP with tIO (*n* = 53; 13.3%), multiple TPs with tIO (*n* = 8, 2.0%), and multiple TPs without tIO (*n* = 66, 16.6%). The incidence of extrahepatic microsphere accumulations was higher in patients treated from a single TP than in patients treated from multiple TPs, irrespective of whether tIOs were performed (Table 5). The differences were not statistically significant.

### 3.5. Alternative Strategy: Could Multiple TPs Have Been Used to Replace tIO?

**Figure 2:**
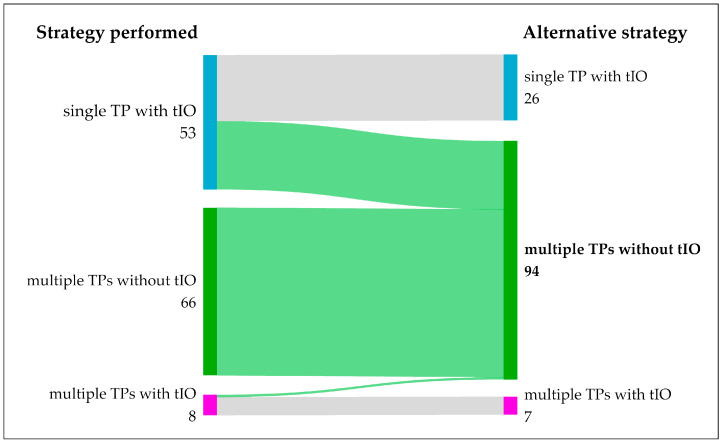
Alternative interventional strategies aiming to inject microspheres from more treatment positions (TPs) while avoiding therapeutic interventional occlusions (tIOs). In 127 TARE procedures, a strategy to avoid extrahepatic microsphere depositions (multiple TPs and/or tIOs) was necessary. Hypothetically, 94 procedures (74.0%) could have been performed without the interventional occlusion of the arteries.

In 27 of 53 single TPs with tIO procedures (50.9%) and in 1 of 8 multiple TPs with tIO procedures (12.5%), TARE treatment would have been possible without the tIO of an extrahepatic vessel if the microspheres were applied from multiple TPs (Figure 2, green flows). In contrast, in 33 procedures (26 single TPs with tIO and 7 multiple TPs with tIO procedures), the tIO was considered obligatory, meaning no alternative with microsphere application outside of multiple TPs would have rendered the tIO unnecessary (Figure 2). Of these cases, 14 TARE treatments required two TPs, and 13 TARE treatments required three TPs. In 19 out of 27 procedures, the avoidance of a tIO would have been possible with more than three TPs and was therefore considered impractical.

In summary, the number of TARE treatments without tIO could have been increased from 337 to 365 procedures (84.7 to 91.7% of overall 398 TARE treatments, respectively).

### 3.6. Alternative Strategy: Would Fewer TPs Have Been Possible by Performing tIO?

**Figure 3:**
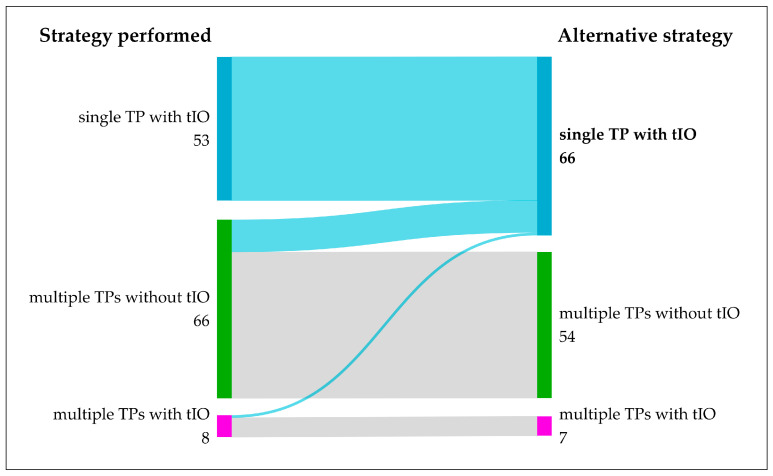
Alternative interventional strategies aiming to inject microspheres from a single treatment position (TP). In 127 TARE procedures, a strategy to avoid extrahepatic microsphere deposition was necessary. Hypothetically, 66 procedures (52.0%) could have been performed from a single TP but with previous interventional occlusions. In 54 procedures, microsphere application from multiple TPs was considered obligatory.

In 12 of 66 multiple TPs without tIO procedures (18.2%) and in 1 of 8 multiple TPs with tIO procedures (12.5%), the alternative application from a single TP with previous tIO could have been used (Figure 3, blue flows). Inversely, in 54 out of 66 cases (81.8%), therapy with multiple TPs could not have been replaced by a single TP with tIO. In 29 procedures, this was due to a complex vascular situation with the potential miss of a tumor-supplying artery due to its origin being close to the origin of the extrahepatic vessel. In 25 procedures, the use of multiple TPs was unavoidable due to two different target areas not being supplied from one common artery.

In eight patients, microsphere application was performed from multiple TPs after a previous tIO. In 6 patients, the combination of both variants was obligatory for successful therapy. In one patient each, therapy could have been achieved by using a single TP after IO or multiple TPs without IO, respectively.

In summary, with tIO, the number of TARE treatments from a single TP could have increased by 13, from 324 to 337 procedures (81.4 to 84.7% of overall 398 TARE treatments, respectively).

## 4. Discussion

Over time, TARE has evolved from a whole-liver approach to more selective treatments, including sub-lobar and segmental interventions. Treatments are tailored to a patient’s needs using voxel-based dosimetry and flexible specific activity per microsphere. Therefore, the interventional radiology approach has also changed from a single proximal injection to multiple catheter positions located deeper into the liver vasculature to ensure that the planned activity is delivered to the desired liver region. This is of particular importance when performing personalized, voxel-based dosimetry to optimize tumor treatment [1].

Non-target accumulations of microspheres into the adjacent, non-hepatic tissues through hepatic-enteric arteries can lead to severe complications, including gastrointestinal ulceration with organ perforation. Therefore, arteries at risk for unwanted microsphere embolization are interventionally occluded preceding TARE with coils, plugs, or foam [4,5]. Alternatively, microsphere application from a position distal to the aberrant vessel without IO is another option [6]. The interventional occlusion of arteries is performed in a variety of settings, including the treatment of acute bleeding and vascular malformations. The choice of embolic material depends on the vascular situation, indication, and availability, but also on the preferences of the interventionalist [5].

### 4.1. Angiographic Complications and Technical Limitations

In our patients, a comparison between TARE evaluation and TARE therapy showed similar incidences of angiographic complications. It was observed that stenoses and vasospasms occurred more frequently during TARE treatment than during TARE evaluation, which might result from repeated irritation of the vessels. In contrast, technical limitations were usually apparent in the first TARE evaluation. In many cases, this was due to the limited knowledge of the hepatic vessel situation before angiography. We recorded coil dislocation, vascular dissections, thrombi, and vasospasms as angiographic complications. Overall, the incidence of vascular-associated complications is similar to other intra-arterial therapies [7]. Another study reported that hepatic arterial complications during radioembolization are mainly associated with coil embolization to prevent non-target delivery to extrahepatic arteries [8].

Both applications from multiple TPs, as well as procedures including IO procedures, did not show a significant additional risk factor for angiographic complications or technical limitations in the patient population studied here. This suggests that the efficient avoidance of extrahepatic microsphere deposition can be ensured without increasing the risk of adverse events (Table 3).

### 4.2. Extrahepatic Microsphere Accumulations

In 398 evaluation angiographies, 69 extrahepatic microsphere accumulations occurred (17.3%), while comparative studies showed higher incidences of up to 31% [9]. In TARE treatment, only a few patients presented with nuclide deposition outside the liver. More recent studies with more experience in TARE emphasize the trend of showing less extrahepatic microsphere deposition after TARE [10]. In more than half of the cases, microsphere depositions were found in the gallbladder and duodenum (Table 4). The low incidence of extrahepatic microsphere accumulation after TARE treatment suggests effective prevention after application from multiple TPs without tIO (1.5%) and after application from one TP with additional tIO (5.3%) (Table 5). This may suggest the superiority of choosing multiple TPs over tIO; however, the difference in the occurrence of microspheres was not statistically significant when comparing the methods (*p* > 0.05).

In a systematic review, Borggreve et al. judged that the occlusion of non-liver vessels is obligatory should the origin be close to an arterial branch and not allow distal TPs [11]. Furthermore, in previous studies, an application from two or three tumor arteries was considered risky and insufficient and subsequently was not evaluated in more detail [11,12]. In contrast, our results suggest that microsphere applications from multiple TPs distal to an aberrant artery can safely avoid extrahepatic enhancement. This agrees with a study comparing 34 TARE therapies with applications proximal to the cystic artery to 31 applications distal to the cystic artery, where 12 and 4 extrahepatic microsphere accumulations could be observed, respectively. The distal injection of the tracer was found to be 2.5 times safer than the proximal injection [12].

In our study, the application of multiple TPs distal to prophylactically occluded vessels foreign to the liver proved to be particularly safe. It was applied distal to a vessel at risk, with the added safety of vessel occlusion in the case of reflux. Despite the low number of only eight procedures, the effective avoidance of extrahepatic microsphere deposition was imaginable when two safe avoidance strategies were combined (Table 5). To assess the clinical relevance, the application in complicated, high-risk patients should be further evaluated.

Extrahepatic microsphere accumulation occurred after approx. 5% of TARE treatments from one TP, regardless of whether IO was performed or not (5.3% and 5.2%, respectively; Table 5). After 74 TARE treatments from more than one TP, extrahepatic microsphere accumulation was detectable in only one case (1.4%; no statistically significant difference due to the small number of cases). It can be assumed that the use of more than one TP does not lead to an increased incidence of extrahepatic microsphere accumulation and may even lead to better prevention than interventions with IO because the TPs are located more distally. Most extrahepatic vessels arise from proximal parts of the hepatic artery, and the risk of microsphere backflow should be lower with a larger distance to their origins [11].

### 4.3. Clinical Adverse Events during Follow-Up

Patients with extrahepatic microsphere accumulations after TARE therapy rarely show clinical symptoms (4 of 18, 22.2%), with most of them being mild or moderate severity (CTCAE < 3). Nevertheless, their prevention is very significant because of the high potential damage they pose. Potentially ischemic cholecystitis (CTCAE < 3) only occurred in the group with the IO of one vessel (cystic artery) (2.8%), showing a less frequent occurrence compared to the literature (3 to 22%) [7,13].

Nevertheless, the overall frequency of adverse events in the 30-day follow-up period after TARE treatment showed no statistically significant difference between the interventional strategies, especially in the comparison of multiple TPs and IO. To our knowledge, this comparison has not yet been made in any previously published study. Nausea and vomiting, as well as upper abdominal discomfort, occurred more frequently after TARE therapy with IO compared to multiple TPs, albeit this was not statistically significant. This may be due to the variably pronounced ischemia of the downstream tissue after IO but may require further structured assessments by questionnaires at predefined intervals for a more systematic evaluation. The investigations of complications and side effects and the comparison with the literature showed similar results [7,14,15]. Overall, adverse events after TARE therapy are mostly of low significance with rare but serious consequences. Future studies should focus on complications and side effects in association with extrahepatic microsphere deposition and different enrichment avoidance strategies.

### 4.4. Indications for Performing Prophylactic Interventional Occlusions

Traditionally, TARE therapy is performed with the prophylactic IO of extrahepatic arteries, which requires a considerable amount of material and time during the intervention and does not show clinical advantage in terms of extrahepatic microsphere accumulations. This was probably due to the increased complexity of the procedure associated with increased radiation exposure, increased vascular injury, and the development of new vascular collaterals, which are again at risk for microsphere embolization [6]. In this context, in 42 patients with therapeutic IO, new non-liver (inferior phrenic artery, intercostal artery, mammary artery, gastro-omental artery) arteries were discovered in 19.7% of cases [16]. The spontaneous recanalization of an IO can also lead to an undesirable microsphere deposition. Half of the patients showed new collaterals, and another 10% showed reopened arteries [17]. This can be explained by altered flow conditions due to occlusion and the opening of previously poorly perfused arterial vessels. Consequently, the adjustment of the catheter position or further interventional occlusions may be required before TARE treatment. This can lead to the nonperformance of TARE in a small proportion of patients (11 of 19 patients with newly identified arterial feeders) [18]. As a conclusion from these findings, applications from a distal catheter position without prophylactic IO of the gastroduodenal artery are preferred [19]. Currently, prophylactic IO is only considered in the presence of a visible retrograde contrast flow or pretreatment with bevacizumab [6]. In our patient cohort, only four patients underwent prophylactic IO of the right gastric artery or gastroduodenal artery.

The following aspects could be identified for the obligatory use of a prophylactic IO:

- No sufficient safety distance between the catheter position and the artery to be omitted;
- Complex vascular situations requiring the use of more than three TPs, making the procedure too complex for clinical practice;
- Hepatic arteries with a small diameter supplying the tumor, which cannot be probed by a catheter and thus require a more proximal application.

### 4.5. Alternative Use of Interventional Strategies with Multiple TPs or tIO

The post hoc analysis of angiographic images of 66 patients with TARE therapy from multiple TPs without tIO and 53 patients with TARE therapy and tIO showed high flexibility between both methods. In 50.9% of cases with TARE after the previous tIO, applications from multiple TPs would have rendered the tIO unnecessary (Figure 2, green flows). In 18.2% of cases performed with multiple TPs, a tIO would have enabled a TARE from a single TP (Figure 2, blue flows). Overall, an approach with tIO could have been used in half of the cases (66 of 127 procedures) where a deposition avoidance strategy was required. The choice of multiple TPs, on the other hand, would have been possible in over three-quarters of cases (94 of 127 procedures).

Factors identified as possible impediments to using a tIO procedure are as follows:

- Multiple vessels in need of occlusion;
- Arteries into which the catheter cannot be positioned;
- Persistent flow despite occlusion procedure;
- Risk of dislocating the occlusion material during the IO procedure.

Moreover, if the choice of multiple TPs is preferred, the following requirements apply:

- The possibility of bypassing the non-liver artery;
- The inclusion of all vessels requiring treatment (i.e., vessels supplying liver regions with tumors);
- Accessibility of a stable and reproducible catheter position for application.

The decision to choose multiple TPs or to perform tIO and fewer TPs should be based on clinical superiority in terms of low incidence of angiographic complications, side effects, and the safe prevention of extrahepatic microsphere accumulation. In particular, the choice of multiple TPs as a possible alternative to IO procedures should be considered if technically feasible and demands a flexible catheter system with precise control in fine vessels.

A more precise microsphere deposition is a potential advantage of TARE from multiple TPs since similar-looking TPs on angiographic imaging may produce different microsphere flows depending on blood flow velocity, injection speed, and the wall attachment of the catheter tip [20,21]. A distal split in deposition localization can be expected to result in a more reproducible microsphere distribution between planning and treatment due to the reduced influence of the positional and bending variability of the catheter tip. The influence of proximal flow conditions may, therefore, be minimized.

### 4.6. Limitations

Limitations of the study result from the retrospective design. Less severe post-therapeutic adverse events may be underestimated. The aggregation of the patients was consecutive but not randomized to interventional strategies. Alternative interventional strategies were deemed feasible based on the available images. Circumstances such as difficulties in probing certain arteries or dynamic backflows during injection are not known unless explicitly mentioned in the reports. The individual personal experience and preferences of treating radiologists and nuclear medicine physicians may also have influenced how procedures were performed and which strategies were used. For a clear comparison of a traditional TARE (single TPs with or without tIO) with microsphere application from multiple TPs, both methods should have been equally valid alternatives, but in clinical reality, technical options are limited due to the individual vascular situation (e.g., localization of the extrahepatic vessels in relation to hepatic arteries) of the patient.

## 5. Conclusions

Our findings indicate that complications due to extrahepatic microsphere depositions, though rare, can result in serious adverse events, underscoring the importance of strategies to prevent these depositions. Utilizing multiple catheter positions during TARE is a feasible and safe approach to mitigate the risk of tracer deposition outside the liver and can, in many cases, replace interventional occlusions of extrahepatic arteries. The use of multiple TPs did not increase the rate of angiographic complications. Moreover, employing multiple TPs enhances the accuracy of dose deposition within the liver. This more selective approach should be integrated into angiographic interventional strategies, particularly in cases involving aberrant vessels, to optimize therapeutic outcomes in TARE procedures. Consequently, our results advocate for the consideration of multiple TPs in the interventional decision-making process to improve the safety and efficacy of TARE.

## Figures and Tables

**Table 1:** Characteristics of interventional occlusions (IO) prior to TARE treatments.

Extrahepatic Artery	Therapeutic IO (*n* = 61)	Prophylactic IO (*n* = 18)
cystic artery	53 (86.9%)	10 (55.5%)
phrenic branches	2 (3.3%)	0
gastric branches	2 (3.3%)	3 (16.6%)
falciform artery	2 (3.3%)	0
duodenal branches	2 (3.3%)	3 (16.6%)
accessory arteries	0	2 (11.1%)

**Table 2:** Indications for microsphere application from multiple treatment positions.

Indication	TARE Treatments (*n* = 398)
(potential) extrahepatic microsphere accumulation or flow to non-target liver	28 (7.0%)
treatment of target volumes without common supplying artery	25 (6.3%)
target volume supplied from non-liver arteries (e.g., left gastric artery and superior mesenteric artery)	14 (3.5%)
unpredictable distribution of microspheres at vascular bifurcations	7 (1.8%)

**Table 3:** Angiographic adverse events and technical limitations.

		TARE Evaluation (*n* = 438)	TARE Treatment (*n* = 398)
Adverse event		10 (2.3%)	7 (1.8%)
	inguinal hematoma	5 (1.1%)	2 (0.6%)
	coil displacement	3 (0.7%)	-
	pseudoaneurysm	1 (0.2%)	1 (0.2%)
	dissection	1 (0.2%)	2 (0.6%)
	circulatory dysregulation	-	1 (0.2%)
	self-limiting hemorrhage from hepatic artery branch	-	1 (0.2%)
Technical limitation		11 (2.5%)	5 (1.2%)
	reversible vasospasm	3 (0.7%)	2 (0.6%)
	TP vessel not accessible	1 (0.2%)	1 (0.2%)
	CA occlusion not feasible	5 (1.1%)	-
	vascular stenosis	2 (0.5%)	2 (0.6%)

TP, treatment position; CA, cystic artery.

**Table 4:** Characteristics of extrahepatic microsphere accumulations after TARE therapy.

Criterion		*n* (%)
Localization, *n* (%)	gallbladder	7 (38.8%)
	duodenum	6 (33.3%)
	stomach	2 (11.1%)
	lymph nodes/connective tissue	2 (11.1%)
	esophagus	1 (5.6%)
Intensity, *n* (%)	above liver level	8 (44.4%)
	at liver level	3 (16.6%)
	below liver level	7 (38.8%)
Microspheres type, *n* (%)	^90^Y-resin	4 (22.2%)
	^90^Y-glass	14 (77.8%)
	^166^Ho-PLLA	0 (0.0%)
Related symptoms, *n* (%)	none	14 (77.8%)
	CTCAE < 3	3 (16.7%)
	CTCAE ≥ 3	1 (5.6%)

**Table 5:** Incidence of extrahepatic microsphere accumulation with different interventional strategies.

Interventional Strategy	Extrahepatic Microsphere Depositions
Single TP without tIO (*n* = 271)	14 (5.2%)
Single TP with tIO (*n* = 53)	3 (5.3%)
Multiple TP without tIO (*n* = 66)	1 (1.5%)
Multiple TP with tIO (*n* = 8)	0
All treatments (*n* = 398)	18 (4.5%)

## Data Availability

The original contributions presented in the study are included in the article; further inquiries can be directed to the corresponding authors.
